# Dermoscopy-guided sampling improves the diagnostic yield of fungal culture for onychomycosis: a comparative study

**DOI:** 10.3389/fmed.2025.1703199

**Published:** 2026-01-02

**Authors:** Bingshen Guo, Yanzhi Bai, Na Qin, Yanling Li, Rong Rong, Liqian Luo, Yuying Zhang, Ruimin Wang

**Affiliations:** Department of Dermatology, The Second Hospital of Hebei Medical University, Shijiazhuang, Hebei, China

**Keywords:** onychomycosis, dermoscopy, fungal culture, diagnostic accuracy, sampling technique

## Abstract

**Background:**

The diagnosis of onychomycosis relies on mycological examination, but the high false-negative rate of fungal culture, often due to improper sampling, remains a significant clinical challenge. Dermoscopy has emerged as a valuable tool for visualizing specific nail structures associated with fungal invasion. This study aimed to evaluate whether using dermoscopy to guide the site of specimen collection can improve the positive rate of fungal culture for onychomycosis compared to conventional sampling.

**Methods:**

We conducted a comparative study involving 1,843 nail units from patients with clinically suspected onychomycosis. Samples were allocated to either a conventional sampling group (*n* = 791) or a dermoscopy-guided sampling group (*n* = 1,052). In the guided group, specimens were collected from sites exhibiting specific dermoscopic features indicative of fungal presence, such as spikes, ruin-like patterns, and longitudinal striations. The primary outcome was the fungal culture positive rate. Secondary outcomes included diagnostic performance metrics, the association between dermoscopic features and culture positivity, and subgroup analyses.

**Results:**

The dermoscopy-guided group demonstrated a significantly higher fungal culture positive rate than the conventional group (72.5% vs. 66.0%, *P* < 0.0001). Within the guided group, dermoscopic features like “spikes” (OR 5.31, 95% CI 4.23–6.66), “ruin-like” appearance (OR 4.31, 95% CI 2.59–7.18), and “longitudinal striations” (OR 2.21, 95% CI 1.76–2.78) were strong predictors of a positive culture (all *P* < 0.001). Multivariate logistic regression confirmed that dermoscopy-guided sampling was an independent predictor of a positive culture (Adjusted OR 1.63, 95% CI 1.30–2.04) after adjusting for confounders. The contamination rate in the guided group was low at 1.4%, and the predominant pathogen identified was *Trichophyton rubrum* (75.2% of positive cultures).

**Conclusion:**

Dermoscopy-guided sampling significantly improves the diagnostic yield of fungal culture for onychomycosis by enabling precise targeting of viable fungal reservoirs. Integrating dermoscopy into the routine sampling procedure is a simple, non-invasive, and effective strategy to reduce false-negative results and enhance diagnostic accuracy.

## 1 Introduction

Onychomycosis is the most common nail disorder encountered in clinical practice, accounting for approximately 50% of all onychopathies and affecting a significant portion of the global population (1–3). It is primarily caused by dermatophytes, but yeasts and non-dermatophyte molds can also be causative agents (1, 4). Beyond being a cosmetic concern, onychomycosis can lead to pain, physical impairment, and a reduced quality of life. In immunocompromised individuals, it can serve as a portal for severe secondary bacterial infections (5). Therefore, accurate diagnosis is crucial for initiating appropriate and often lengthy antifungal therapy, thereby preventing treatment failure and the development of drug resistance (6).

The gold standard for diagnosing onychomycosis involves the combination of direct microscopy [typically with potassium hydroxide (KOH)] and fungal culture (7, 8). While microscopy offers rapid detection of fungal elements, it cannot identify the species or confirm viability. Fungal culture is essential for species identification, which can guide therapeutic decisions and provide epidemiological insights, but it is hampered by a notably high false-negative rate, reported to be over 35% in some studies (9, 10). This diagnostic gap is largely attributed to improper specimen collection, where the sample may contain insufficient fungal load, non-viable organisms, or contaminants (6).

Dermoscopy, a non-invasive *in vivo* imaging technique, has revolutionized the diagnosis of various dermatological conditions by allowing visualization of subsurface structures (11). In recent years, its application has expanded to nail disorders, including onychomycosis (9, 12). Several characteristic dermoscopic patterns have been associated with onychomycosis, such as jagged or “spiky” proximal edges, longitudinal striations, a “ruin-like” or pulverized appearance at the distal edge, and chromonychia (6, 9, 13). These features correspond to areas of active fungal invasion within the nail unit (8).

While dermoscopy is increasingly recognized as an auxiliary diagnostic tool (6, 14), its potential to improve mycological test yield by guiding sampling is not yet well-established through large-scale evidence. Traditional sampling relies on naked-eye assessment to scrape debris from affected subungual areas, a subjective method that may miss viable fungal reservoirs (6). We hypothesized that using dermoscopy to pinpoint features indicative of fungal presence (e.g., jagged edges, ruin patterns) would enable targeted sampling, increasing fungal culture positivity (6, 13, 14).

This study aimed to formally test this hypothesis by comparing the fungal culture positive rates between a conventional, visually-guided sampling method and a dermoscopy-guided sampling technique in a large cohort of patients with clinically suspected onychomycosis. We also sought to identify which specific dermoscopic features are most strongly correlated with a positive culture result, providing an evidence-based framework for this enhanced diagnostic approach.

## 2 Materials and methods

### 2.1 Study design and population

This comparative study was conducted at the Department of Dermatology, the Second Hospital of Hebei Medical University, between January 2021 and December 2021. The study was designed and reported following the Standards for Reporting of Diagnostic Accuracy Studies (STARD) 2015 guidelines (15). The study protocol was approved by the Institutional Ethics Committee of the Second Hospital of Hebei Medical University, and written informed consent was obtained from all participants or their legal guardians prior to enrollment.

We enrolled patients presenting with a clinical suspicion of onychomycosis. Inclusion criteria were: (1) clinical signs suggestive of onychomycosis, such as nail discoloration, subungual hyperkeratosis, onycholysis, or nail plate dystrophy; and (2) willingness to undergo all study procedures, including dermoscopy, specimen collection, and mycological examinations. Exclusion criteria were: (1) systemic antifungal treatment within the last 3 months or topical antifungal treatment within the last 2 weeks; and (2) concomitant nail diseases that could mimic onychomycosis, such as nail psoriasis, lichen planus, or traumatic onychodystrophy, as determined by a senior dermatologist.

Enrolled nail units were non-randomly allocated into two groups based on the sampling procedure available at the time of their visit: the conventional sampling group and the dermoscopy-guided sampling group.

### 2.2 Data collection and clinical assessment

For each included nail unit, we recorded baseline demographic data (age, gender), clinical information (location of the affected nail: fingernail or toenail), and the clinical subtype of onychomycosis. Clinical subtypes were classified according to established criteria into distal and lateral subungual onychomycosis (DLSO), superficial white onychomycosis (SWO), proximal subungual onychomycosis (PSO), and total dystrophic onychomycosis (TDO) (16).

### 2.3 Specimen collection procedures

#### 2.3.1 Conventional sampling group

In the conventional group, specimen collection followed the recommendations from the Chinese Guidelines for the Diagnosis and Treatment of Onychomycosis (2015 Edition) (17). After cleaning the nail with 75% alcohol, a sterile scalpel or curette was used to collect subungual debris and nail clippings from the most dystrophic-appearing area at the junction of the affected and healthy nail plate, as identified by the naked eye.

#### 2.3.2 Dermoscopy-guided sampling group

In the dermoscopy-guided group, a dermatologist trained in onychoscopy first examined the affected nail using a handheld dermatoscope (BN-PFMF-8001, Nanjing Benin Medical Equipment Co., Ltd., China) in polarized mode with 10× magnification. The presence of specific dermoscopic features was recorded, including: (1) Spikes: A jagged, spiked, or serrated pattern at the proximal border of onycholysis; (2) Longitudinal striations: Linear bands of color (typically white, yellow, or orange) extending from the distal edge toward the proximal nail; and (3) Ruin-like appearance: A pulverized, crumbly, and heterogeneous pattern of the distal nail edge (12, 18).

After identifying the most prominent and accessible of these features, the same location was marked. The nail was then cleaned with 75% alcohol, and a sterile scalpel was used to precisely collect subungual material directly from the marked site under dermoscopic guidance.

### 2.4 Mycological examination

All collected specimens were divided into two parts for fluorescence microscopy and fungal culture.

#### 2.4.1 Fluorescence microscopy

One portion of the sample was placed on a glass slide, and a commercial fluorescent staining kit (based on calcofluor white) was added. After a 5–10 min incubation period, the slide was examined under a fluorescence microscope (Leica DM500, Germany). The presence of bright green fluorescent hyphae and/or spores was considered a positive result.

#### 2.4.2 Fungal culture

The other portion of the specimen was inoculated onto Sabouraud Dextrose Agar (SDA) plates, with and without cycloheximide and chloramphenicol, using a multi-point inoculation technique. The plates were incubated at 25 °C–28 °C for up to 3 weeks and checked regularly for growth. A culture was considered positive if a dermatophyte was isolated. For yeasts or non-dermatophyte molds, positivity was confirmed if the same organism grew from at least 6 out of 10 inoculation points and the microscopy showed corresponding fungal elements, as per established laboratory criteria (19). Species identification was performed based on colony morphology and microscopic features of lactophenol cotton blue mounts (20). Contamination was recorded based on laboratory remarks (e.g., isolation of common environmental molds like *Aspergillus niger* or *Penicillium* in a scattered pattern).

### 2.5 Statistical analysis

Data were analyzed using SPSS software, version 16.0. Continuous variables (age) were presented as mean ± standard deviation (SD) and compared using the independent samples *t*-test. Categorical variables (gender, clinical type, positive rates) were presented as counts and percentages (*n*, %) and compared using the Chi-square (χ^2^) test or Fisher’s exact test, as appropriate. A *P*-value < 0.05 was considered statistically significant.

For the primary outcome, the fungal culture positive rates between the two groups were compared using the χ^2^ test. In the dermoscopy-guided group, diagnostic performance metrics [sensitivity, specificity, positive predictive value (PPV), negative predictive value (NPV), and accuracy] of culture were calculated using fluorescence microscopy as the reference standard. The association between specific dermoscopic features and culture positivity was assessed using binary logistic regression to calculate odds ratios (ORs) and their 95% confidence intervals (CIs). Finally, a multivariate logistic regression model was constructed to identify independent predictors of a positive culture, including the sampling group, age, gender, and clinical subtype as variables.

## 3 Results

### 3.1 Baseline characteristics of the study population

**FIGURE 1:**
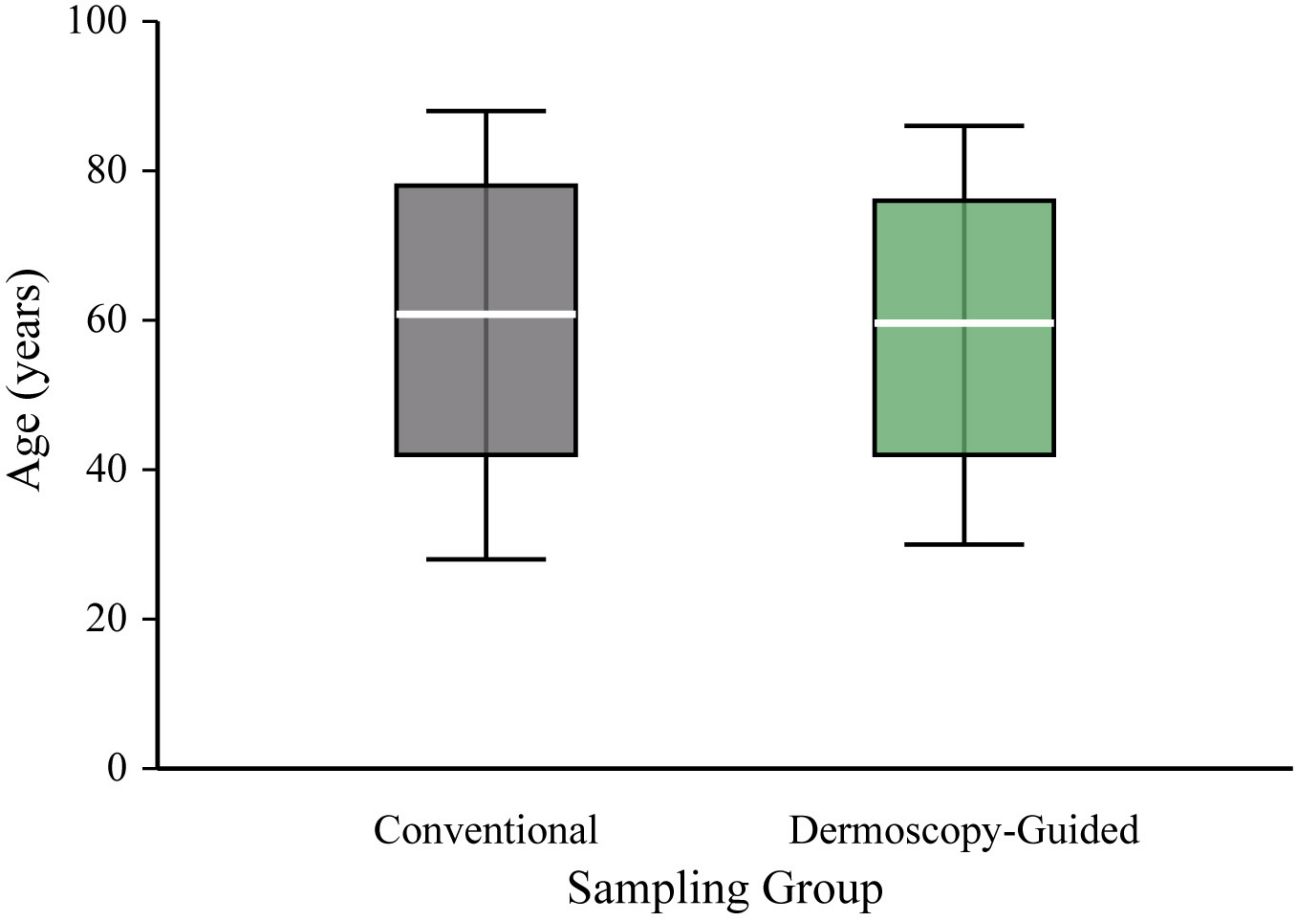
Boxplot of age distribution between the conventional and dermoscopy-guided groups. The boxes represent the interquartile range (IQR), the horizontal line inside the box represents the median, and the whiskers extend to 1.5 times the IQR. No significant difference was observed between the two groups (*P* = 0.512).

A total of 1,843 nail units from clinically suspected onychomycosis patients were included in this study. Of these, 791 were assigned to the conventional sampling group and 1,052 to the dermoscopy-guided sampling group. The baseline demographic and clinical characteristics of the samples were well-balanced between the two groups. There were no statistically significant differences in terms of mean age (41.5 ± 19.8 vs. 40.9 ± 18.5 years, *P* = 0.512), gender distribution (38.0% vs. 38.3% male, *P* = 0.835), or the distribution of clinical subtypes (*P* > 0.999). Detailed baseline characteristics are presented in Table 1. The similarity in age distribution between the two groups is also visually confirmed in the boxplot in Figure 1.

**TABLE 1:** Baseline demographic and clinical characteristics of nail units in the two groups.

Characteristic	Conventional group (*n* = 791)	Dermoscopy-guided group (*n* = 1052)	*P*-value
Age (years, Mean ± SD)	41.5 ± 19.8	40.9 ± 18.5	0.512^a^
Gender, *n* (%)			0.835^b^
Male	301 (38.0)	403 (38.3)	
Female	490 (62.0)	649 (61.7)	
Clinical type, *n* (%)			>0.999^b^
DLSO	413 (52.2)	550 (52.3)	
SWO	213 (26.9)	282 (26.8)	
TDO	140 (17.7)	185 (17.6)	
PSO	25 (3.2)	35 (3.3)	

SD, standard deviation; DLSO, distal and lateral subungual onychomycosis; SWO, superficial white onychomycosis; TDO, total dystrophic onychomycosis; PSO, proximal subungual onychomycosis.

^a^Calculated by independent samples *t*-test.

^b^Calculated by Chi-square test.

### 3.2 Comparison of fungal culture positive rates and diagnostic performance

The primary outcome of the study was the fungal culture positive rate. As shown in Table 2, the dermoscopy-guided sampling group achieved a significantly higher positive rate compared to the conventional group (72.5% vs. 66.0%, *P* < 0.0001). To further assess the diagnostic performance within the dermoscopy-guided group, we compared the culture results against fluorescence microscopy (KOH) as a reference standard (Table 3). The dermoscopy-guided culture method demonstrated a sensitivity of 77.0% and a specificity of 46.5%. Notably, a positive culture was obtained in 106 cases that were negative by microscopy, highlighting its ability to detect viable fungi potentially missed by direct examination.

**TABLE 2:** Overall comparison of fungal culture positive rates between the two groups.

Group	Total nails	Positive, *n*	Negative, *n*	Positive rate (%)	χ ^2^ value	*P*-value
Conventional group	791	522	269	66.0	49.38	<0.0001
Dermoscopy-guided group	1052	763	289	72.5		

**TABLE 3:** Diagnostic performance of dermoscopy-guided culture versus fluorescence microscopy in the dermoscopy-guided group (*n* = 1052).

Dermoscopy-guided culture	Fluorescence microscopy (KOH)		Total
	Positive	Negative	
Positive	657 (TP)	106 (FP)	763
Negative	197 (FN)	92 (TN)	289
Total	854	198	1052

Key metrics: Sensitivity = 77.0%; Specificity = 46.5%; PPV = 86.1%; NPV = 31.8%; Accuracy = 71.2%. TP, True Positive; FP, False Positive; FN, False Negative; TN, True Negative; PPV, positive predictive value; NPV, negative predictive value.

### 3.3 Correlation of dermoscopic features with culture positivity

To elucidate the mechanisms underlying the improved yield of the guided method, we analyzed the association between specific dermoscopic features and culture positivity within the dermoscopy-guided group (Table 4). Among all observed features, the presence of “spikes” was identified as the most powerful predictor of a positive culture, increasing the odds by over fivefold (OR 5.31, 95% CI 4.23–6.66; *P* < 0.001). Other significant predictors included a “ruin-like” appearance (OR 4.31, 95% CI 2.59–7.18; *P* < 0.001) and “longitudinal striations” (OR 2.21, 95% CI 1.76–2.78; *P* < 0.001). These findings provide an evidence-based guide for selecting the optimal sampling site. Furthermore, analysis of the laboratory remarks revealed a very low contamination rate of 1.4% (15/1052), suggesting that precise targeting minimizes the collection of environmental molds.

**TABLE 4:** Association between dermoscopic features and culture positivity in the dermoscopy-guided group (*n* = 1052).

Dermoscopic feature	Positive culture (*n* = 763)	Negative culture (*n* = 289)	Odds ratio (95% CI)	*P*-value
Spikes, *n* (%)	526 (69.0%)	78 (27.0%)	5.31 (4.23–6.66)	<0.001
Ruin-like, *n* (%)	82 (10.7%)	10 (3.5%)	4.31 (2.59–7.18)	<0.001
Longitudinal striations, *n* (%)	508 (66.6%)	129 (44.6%)	2.21 (1.76–2.78)	<0.001

Of the 1285 total positive cultures across both groups, species identification was successful in 1268 cases (98.7%). The distribution of fungal isolates is detailed in Supplementary Table 1 and visualized in Supplementary Figure 1. Dermatophytes were the most common pathogens, accounting for 89.1% of isolates, followed by yeasts (7.3%) and non-dermatophyte molds (3.6%). Among dermatophytes, *Trichophyton rubrum* was overwhelmingly the most frequent species (75.2% of all isolates), consistent with its known global prevalence in onychomycosis (21).

### 3.4 Subgroup analyses and predictors of positive culture

**FIGURE 2:**
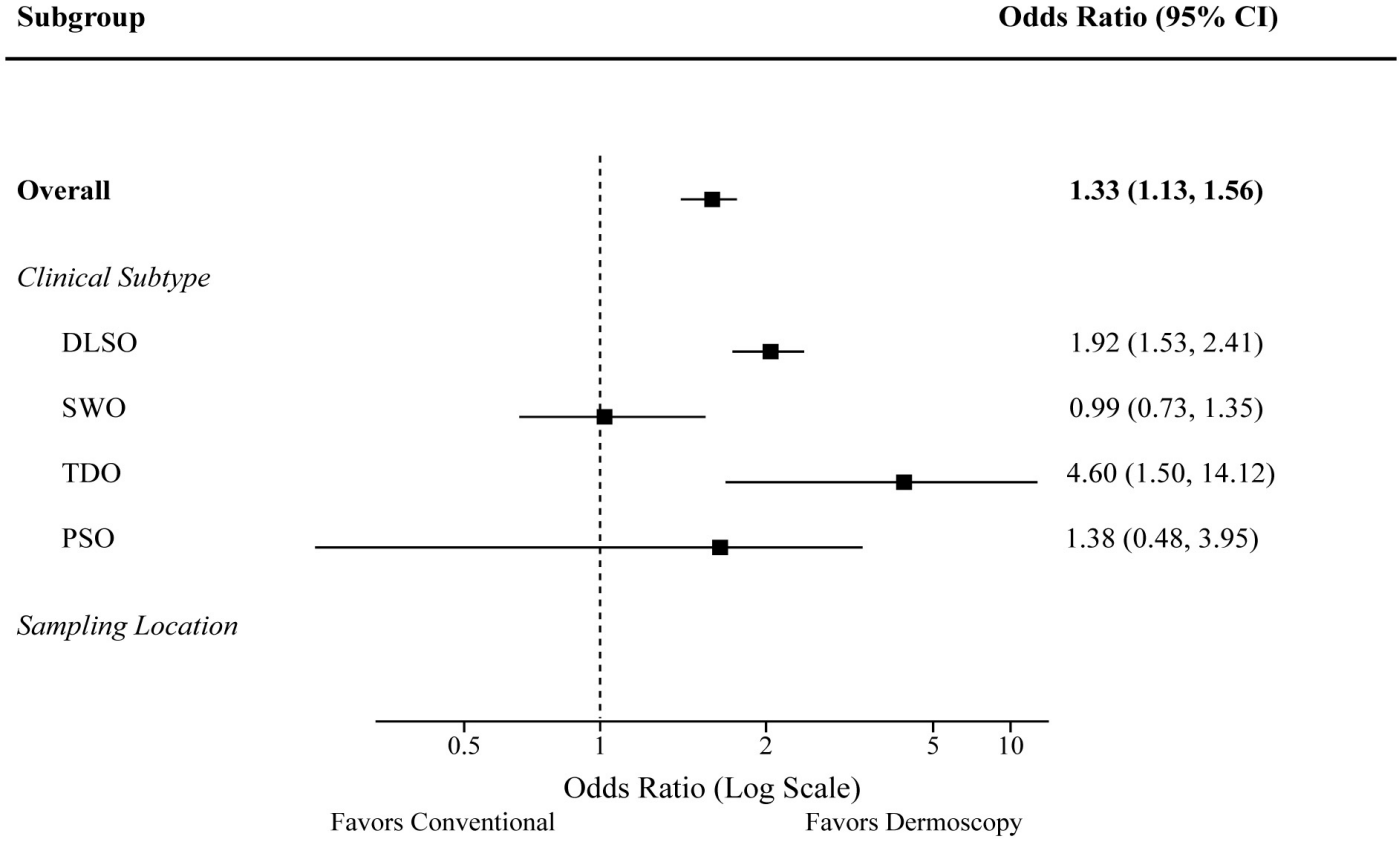
Forest plot of subgroup analyses for the odds of a positive fungal culture. The squares represent the odds ratio (OR), and the horizontal lines represent the 95% confidence intervals (CI). An OR greater than 1 favors the dermoscopy-guided group.

Subgroup analyses were performed to further investigate the efficacy of dermoscopy-guided sampling across clinical subtypes and sampling locations. The forest plot in Figure 2 visualizes the odds ratios (ORs) for a positive culture in the dermoscopy-guided group compared to the conventional group. The benefit of dermoscopy guidance was most pronounced in the DLSO subtype (OR 1.92, 95% CI 1.53–2.41) and the TDO subtype (OR 4.60, 95% CI 1.50–14.12). When stratified by location, the advantage was significant for both toenails and fingernails, as detailed in Table 5.

**TABLE 5:** Subgroup analysis of fungal culture positive rates by sampling location.

Location	Group	Total nails (*n*)	Positive (*n*)	Positive rate (%)	*P*-value
Fingernails	Conventional	220	140	63.6	0.041
	Dermoscopy-guided	301	210	69.8	
Toenails	Conventional	571	382	66.9	0.011
	Dermoscopy-guided	751	553	73.6	

To identify independent predictors for a positive culture, a multivariate logistic regression analysis was performed on the entire cohort. As shown in Table 6, after adjusting for potential confounders including age, gender, and clinical subtype, dermoscopy-guided sampling remained a strong and independent predictor of a positive culture (Adjusted OR 1.63, 95% CI 1.30–2.04, *P* < 0.001). Additionally, the TDO clinical subtype was found to be strongly associated with a higher likelihood of a positive result compared to the DLSO subtype (Adjusted OR 8.51, 95% CI 3.71–19.51, *P* < 0.001).

**TABLE 6:** Multivariate logistic regression analysis of predictors for positive fungal culture.

Variable	Adjusted odds ratio (aOR)	95% Confidence interval (CI)	*P*-value
Sampling group (dermoscopy vs. conventional)	1.63	1.30–2.04	<0.001
Age (per year increase)	1.00	0.99–1.01	0.815
Gender (female vs. male)	0.95	0.76–1.19	0.654
**Clinical subtype (vs. DLSO)**			
SWO	1.02	0.77–1.34	0.902
TDO	8.51	3.71–19.51	<0.001
PSO	0.71	0.36–1.41	0.330

The model was adjusted for all variables listed in the table. The reference categories are conventional group for sampling group, male for gender, and DLSO for clinical subtype.

## 4 Discussion

The accurate diagnosis of onychomycosis is fundamental to its successful management, yet the standard diagnostic pathway is often undermined by the low sensitivity of fungal culture, which can be prone to false-negative results (9, 22). Our study robustly demonstrates that integrating dermoscopy to guide specimen collection is a highly effective strategy to overcome this long-standing limitation. The central finding of our research is that dermoscopy-guided sampling significantly increases the positive rate of fungal culture compared to the conventional method (72.5% vs. 66.0%). This result, supported by a large sample size, provides strong evidence for the superiority of a targeted sampling approach.

The enhanced yield is likely attributable to the ability of dermoscopy to identify specific subclinical features that correlate with high concentrations of viable fungi. Our analysis of dermoscopic patterns within the guided group provides a mechanistic explanation for this success. The “spikes” pattern emerged as the most powerful predictor of a positive culture, increasing the odds by more than five times. This finding is consistent with previous histopathological correlations, which suggest that these jagged edges represent the advancing front of fungal invasion, where fungal hyphae are actively degrading the nail keratin (14, 23). Similarly, the “ruin-like” appearance and “longitudinal striations” were also potent indicators. These patterns likely correspond to areas of significant nail plate destruction and linear fungal colonization, respectively, making them ideal targets for sampling (8, 24). By providing clinicians with a clear, evidence-based roadmap of what to target, dermoscopy transforms sampling from a subjective estimation into a precise, targeted procedure.

An interesting secondary finding from our analysis was the very low contamination rate of 1.4% in the dermoscopy-guided group. Although we did not have contamination data for the conventional group to perform a direct comparison, this low rate suggests another potential benefit of the guided technique. Precise targeting may minimize the collection of superficial, non-pathogenic environmental molds and bacteria that can contaminate the culture plate and obscure or inhibit the growth of true pathogens (13). This contributes to a more reliable and interpretable culture result.

Our subgroup analyses further reinforce the value of the dermoscopy-guided approach across different clinical scenarios. The technique provided a significant benefit for both fingernails and toenails and was particularly advantageous for the DLSO (distal and lateral subungual onychomycosis) and TDO (total dystrophic onychomycosis) subtypes. TDO, characterized by extensive nail destruction, can be a challenging subtype for conventional sampling, as it is difficult to distinguish viable fungal areas from necrotic debris. Dermoscopy appears to be especially useful in these cases for locating residual pockets of active infection. Specific patterns are often associated with certain subtypes; for instance, DLSO commonly presents with “spikes” and “longitudinal striations,” while TDO frequently exhibits a “distal irregular termination” pattern (24–26). The multivariate analysis confirmed that the benefit of dermoscopy-guided sampling is not merely a reflection of other factors but is an independent predictor of diagnostic success. The strong association of the TDO subtype with culture positivity itself (aOR 8.51) is expected, given the extensive fungal burden in this presentation.

When evaluating the diagnostic performance of the guided culture method against microscopy, we observed a moderate sensitivity (77.0%) but a low specificity (46.5%). The low specificity, indicated by the 106 culture-positive but microscopy-negative cases, is particularly noteworthy. It suggests that culture can detect viable fungi even when the fungal load is too low to be readily seen on a direct smear or when the sample contains primarily arthroconidia, which can be harder to identify microscopically (25, 27). This highlights a key strength of our targeted culture approach: its ability to capture viable organisms that might otherwise be missed, leading to a false-negative diagnosis if relying on microscopy alone.

Despite the improvement with dermoscopy guidance, a notable false-negative rate remained (27.5% in the guided group). This highlights the inherent limitations of fungal culture as a diagnostic tool. Several factors may contribute to this residual false-negative rate. First, culture requires viable organisms, and samples may contain non-viable fungal elements that are detectable by microscopy but fail to grow (28). Second, some fungal species are fastidious and may not grow on standard SDA medium, or their growth may be inhibited by faster-growing contaminants despite careful sampling. Third, even with precise guidance, the sampled material may originate from an area with low fungal density or from a deep-seated focus that is inaccessible to a superficial scraping. Finally, undeclared prior use of topical antifungal agents by patients remains a possibility that can suppress fungal viability. These factors underscore that while dermoscopy-guided sampling optimizes the quality of the specimen, it cannot overcome the intrinsic biological and technical challenges of the culture method itself. The future of onychomycosis diagnosis may therefore lie in combining dermoscopy-guided sampling with more sensitive molecular techniques, such as fungal PCR, to further close this diagnostic gap (22, 29, 30).

This study has several strengths, including its large sample size, comparative design, and detailed analysis of dermoscopic predictors. However, some limitations should be acknowledged. Firstly, the allocation to the two groups was not randomized, which could introduce selection bias. However, the baseline characteristics of the two groups were remarkably similar, suggesting that this bias was likely minimal. Secondly, our reference standard for calculating diagnostic metrics was fluorescence microscopy, not a more definitive standard like histopathology or PCR. Nevertheless, microscopy is the most widely used comparator in routine clinical practice. A further limitation is that contamination rates were not systematically recorded for the conventional group in this study, which precluded a direct statistical comparison between the two methods, although the observed rate was low. Future research could incorporate molecular methods to further validate these findings.

## 5 Conclusion

In conclusion, our study provides compelling evidence that using dermoscopy to guide specimen collection significantly improves the diagnostic yield of fungal culture for onychomycosis. The identification of specific dermoscopic features—particularly spikes, a ruin-like appearance, and longitudinal striations—as strong predictors of culture positivity equips clinicians with a practical, evidence-based tool to enhance sampling accuracy. The adoption of this simple, non-invasive technique into routine clinical practice can reduce the high rate of false-negative results, leading to more timely and appropriate treatment for patients with onychomycosis.

## Data Availability

The original contributions presented in this study are included in this article/Supplementary material, further inquiries can be directed to the corresponding author.
