# Supplementary figures and images for: Dermoscopy-guided sampling improves the diagnostic yield of fungal culture for onychomycosis: a comparative study
Source: Front Med (Lausanne). 2026 Jan 2;12:1703199. doi: 10.3389/fmed.2025.1703199 (PMC12807977; doi:10.3389/fmed.2025.1703199)

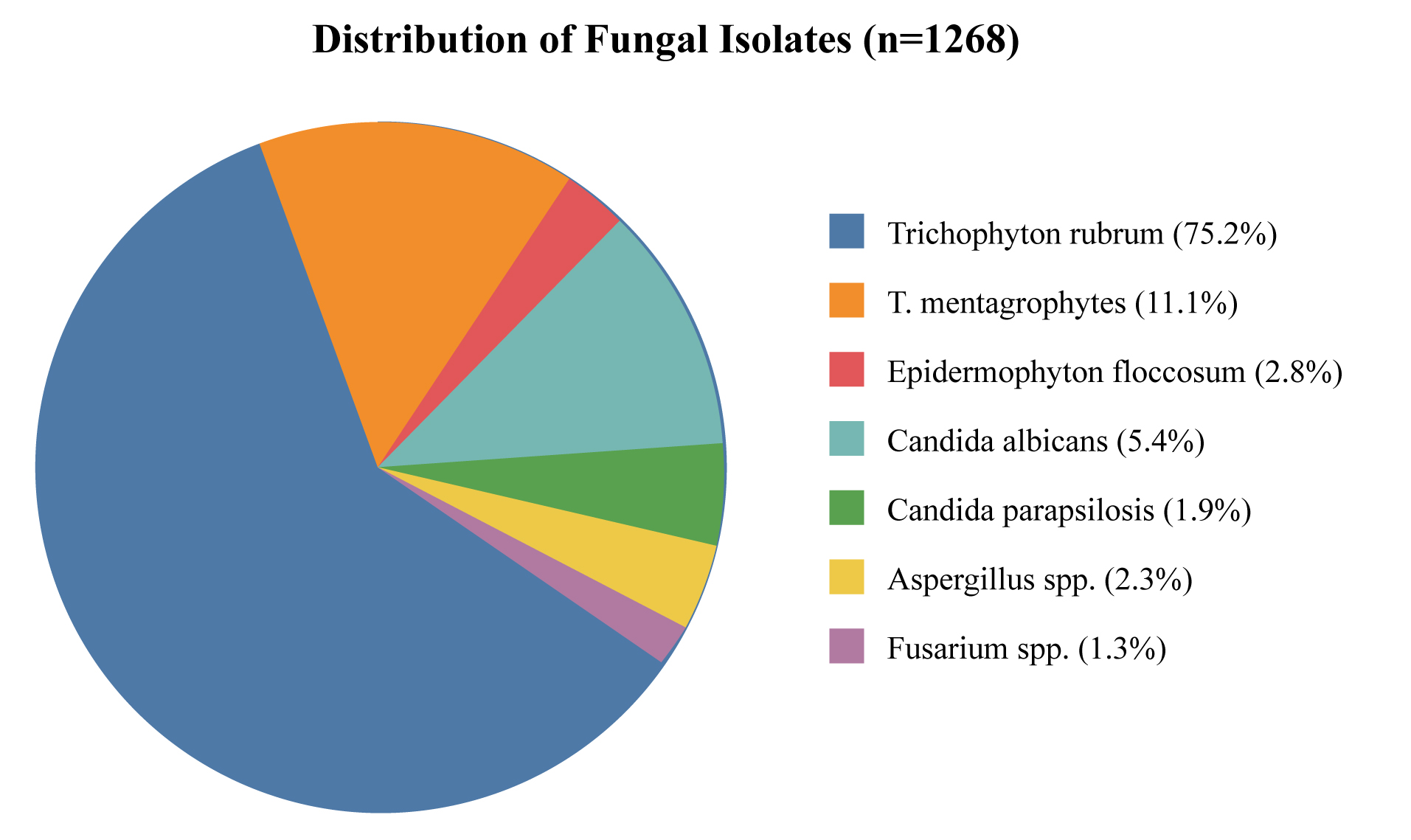

Supplement: Supplementary file 1 [file Image_1.jpeg]
