# Supplementary material for: Dermoscopy-guided sampling improves the diagnostic yield of fungal culture for onychomycosis: a comparative study
Source: Front Med (Lausanne). 2026 Jan 2;12:1703199. doi: 10.3389/fmed.2025.1703199 (PMC12807977; doi:10.3389/fmed.2025.1703199)
Supplement: Supplementary file 2 [file Table_1.docx]

**Supplementary Table S1. Distribution of fungal species identified from positive cultures (n=1268).**

| **Fungal Group / Species** | **Number of Isolates (n)** | **Percentage (%)** |
| --- | --- | --- |
| **Dermatophytes** | **1130** | **89.1** |
| *Trichophyton rubrum* | 953 | 75.2 |
| *Trichophyton mentagrophytes* | 141 | 11.1 |
| *Epidermophyton floccosum* | 36 | 2.8 |
| **Yeasts** | **93** | **7.3** |
| *Candida albicans* | 68 | 5.4 |
| *Candida parapsilosis* | 25 | 1.9 |
| **Non-Dermatophyte Molds (NDMs)** | **45** | **3.6** |
| *Aspergillus* spp. | 29 | 2.3 |
| *Fusarium* spp. | 16 | 1.3 |
| **Total Identified** | **1268** | **100.0** |
